# Inter-provincial inequality of public health services in China: the perspective of local officials’ behavior

**DOI:** 10.1186/s12939-018-0827-8

**Published:** 2018-07-31

**Authors:** Tianxiang Chen, Ying Wang, Xiaoyi Luo, Yuxuan Rao, Lei Hua

**Affiliations:** 1 School of Government, Sun Yat-sen University, Guangzhou, Guangdong China; 2 Department of Public Administration, Nanfang College of Sun Yat-sen University, Guangzhou, Guangdong China; 3 School of Law and Business, College of Science & Technology of Ningbo University, Ningbo, Zhejiang China; 4 College of Liberal Art and Science, University of Illinois at Urbana-Champaign, Urbana, Illinois USA

**Keywords:** Inter-provincial public health services inequality, Public health services equalization, Local officials, Political promotion tournament, Fiscal transfer payments, Fiscal self-sufficiency of local governments, China

## Abstract

**Background:**

After economic reform, China experienced rising public health services inequality between the eastern developed and mid-west undeveloped provinces. The fiscal transfer payment system which aims to shape the disparities was considered inefficient. However, there are only a few studies that address the political reason when analyzing the inter-provincial public health services inequality. And the previous studies did not consider a possible non-linear relationship between the fiscal transfer payments and the inter-provincial public health services equalization.

**Methods:**

This paper argues that the local officials’ fanatical pursuit of local economic growth which driven by the *Political Promotion Tournament* and the polarized fiscal self-sufficiency (fiscal capacities) of local governments are responsible for the inter-provincial inequality of public health services and the inefficiency of fiscal transfer payments. By constructing panel threshold regression models with fiscal self-sufficiency of local governments as threshold variable, this study tries to empirically investigate the optimal level of the local governments’ self-sufficiency at which the fiscal transfer payments can effectively promote equalization.

**Results:**

Threshold effects exist between fiscal transfer payments and inter-provincial public health services equalization. The effects on inter-provincial public health services equalization show trends that first increase and then decrease as the fiscal self-sufficiency of local governments increases. And there exist a range of fiscal self-sufficiency between 29.236 and 43.765% or between 28.575 and 45.746% for local governments where the fiscal transfer payments can effectively achieve equalization. Currently, the vast majority of provinces in China remain in the ineffective regime where the fiscal transfer payments are inefficient in shaping inequality.

**Conclusions:**

This paper explains the reason of inequality in public health services and the inefficiency of fiscal transfer payment system from Chinese local officials’ behavior aspect, and try to find out an effective solution by focusing on the local government’s fiscal capacity. The effective way to narrow the inequality is to establish a flexible tax-sharing system to adjust local governments’ fiscal capacities and give local governments with low fiscal self-sufficiency more fiscal resources. The new policy measures recently launched by Chinese central government coincide with our recommendations.

## Background

In 1978, China launched its market-oriented economic reform, under which the society transformed rapidly from a completely planned economy to a market-dominated economy [1]. This transformation, which characterized by abolishing egalitarianism and strengthening market infrastructure, achieved decades of continued economic growth. Nevertheless, it is also this economic transformation that accidentally or deliberately collapsed the widely acclaimed medical health system which provided prevention and primary care to almost every Chinese [2, 3].

Inequality of public health services, along with inequality of public health expenditures and socioeconomic status, are generally considered as a leading cause for inequality of health [4–6]. In 2015, infant mortality and mortality of children under five years old in the west undeveloped region of China was more than three times than those of eastern developed region [7]. And the number of medical clinic visit per person in 2014 in developed provinces such as *Beijing* and *Shanghai* was 9.93 and 10.33, respectively, while that number in undeveloped provinces such as *Guizhou* and *Qinghai* was only 3.71 and 3.87, respectively [8]. Those information shows that residents in developed provinces of China have better public health services than those in undeveloped provinces, which tells that inequality of public health services among provinces has already been a serious issue in China. And once this inequality has spread, complains and sense of unfairness will increased gradually and eventually devastate the basis that the country relies to develop.

The cause of inequality of health service in China is complex and varies not only by region but also over time. Some provinces have been able to escape this problem, while others have not. To find the causes, a majority of existing researches on inequality of health in China focus on the association between socioeconomic factors and health inequality [9–16]. And literature for the effects of socioeconomic factors such as income and education on the inequality of public health services are sufficient and have been well examined [17–23]. Some other researchers concentrate on health policy implementation and health system reform. By analyzing changes in equality of public health services during China’s economic transition [24–29], they try to accumulate experiences and lessons from history [30, 31].

In general, previous studies argued that the individual social-economic characteristics and regional economic development are the principal determinants of inequality in public health services [32], but ignored the importance of China’s unique political system. In fact, political factors cannot be avoided in any China problems given that China is a country with strong political complexion. And for this topic, the Chinese bureaucracy style, which drives local officials to spare no effort in promoting regional economic development and leads public health services to play second fiddle, is neglected [33, 34]. Meanwhile, most of the previous studies were attracted by a single part inequality of public health services, such as urban-rural inequality or the individual differences in China. Inequality of public health services among provinces, which can be used to predict a country’s future development of people’s livelihood, becomes a critical part that lacks references [5, 35–39].

In fact, from the perspective of historical institutionalism, equalization of public health services is a common problem in many countries during the process of national modernization. One useful way to release the inequality is strengthen the role of market mechanisms, at this point the United States provides a good practice template. As early as 1930, the U.S. government was already concerned about the equalization of public health services, and after decades of development, marketization has played an important role in solving the problem of equalization of public health services [40]. Unlike the United States, societies and social organizations in developing countries such as China, are not mature enough to participate in the management of public health affairs, and nor can they provide public health developing funding. Therefore, reducing individual’s spending on public health services and built relatively mature medical insurance system became another way to release the inequality, and that was what Germany had done ever since Prussian government. In Germany, medical social insurance is the main route for medical funding, medical insurance covers 98% of the country’s population. Although this insurance system brings other financial problem in the following decades, it basically guaranteed the coverage of insurance and provided every individual an access to health service, thus promoting equalization of health services in the state [41]. Nevertheless, this approach could not be applied in developing countries like China, either. The relatively low income level and a great number of peasant decide that there is no supportable tax revenue to afford financial expenditure on highly coverage medical insurance system for developing countries. At this point, some countries including China began to imitate Australia, to use fiscal transfer payment as a basic means to promote its equalization of public health services, will this approach be effective in China?

In 1994, China began to implement a sequence of fiscal transfer payment system, which is essentially a system to reallocate fiscal resources and equalize basic public services including public health service, within regions with different public financial capabilities [42, 43]. However, research performed by most scholars suggest that China’s current fiscal transfer payment system cannot promote inter-provincial equalization of public services efficiently, and the policy suggestions proposed by prior literature neglect the important role of fiscal transfer payment in shaping the disparities [44–47]. There are only a few studies that address the political reason when analyzing the inefficiency of fiscal transfer payment system. More importantly, the previous conclusions about the inefficiency of fiscal transfer payment system were mainly based on the linear regression results and neglected the fact that the effect of fiscal transfer payment on public health services equalization may change with the fiscal self-sufficiency of local governments.

In sum, China had tried what Austria had done by using fiscal transfer payments, but it didn’t work well on its equalization of public health services developing process. Since other approaches are not feasible for countries under a stage where economic development is its primary aim, this paper takes *promotion tournament* (refer to economic competition for better career development among governments) and self-sufficiency of local finance into consider, intends to improve the effectiveness of using fiscal transfer payments as an approach on equalizing public health services. Hence, other developing countries that adopt transfer payments method to achieve equalization of their own public health services, could absorb China’s experience on policy formulation and policy implementation. And specifically, our contribution can be illustrated from the following three aspects.

Initially, our findings make great complements for previous literature by explaining the reason of inequality in public health services and the inefficiency of fiscal transfer payment system from Chinese local officials’ behavior. In China, the cadres’ evaluation system rewards and punishes local officials on the basis of their economic performance, which provides lower-level officials a strong incentive to develop the economy and neglect other areas including public services, to obtain political promotion (*Political Promotion Tournament* theory) [33]. The so called *Political Promotion Tournament* phenomenon is particularly problematic in the undeveloped provinces, which may increase inter-provincial gap in public health services. In undeveloped provinces with low fiscal self-sufficiency, large amount of transfer payment funds was misappropriated by local officials and used to promote local economic growth to pursuit their success in *Political Promotion Tournament,* thus leading to the inefficiency use of fiscal transfer payment on public health services development.

Secondly, we find that the effect of fiscal transfer payments on public health services equalization is not linear but shows a trend that first increases and then decreases with the increasing of local governments’ fiscal self-sufficiency.

The third contribution of the study is to propose an effective solution by focusing on the local government’s fiscal capacity. The effective way to narrow the inter-provincial public health services inequality is to establish a flexible tax-sharing system to adjust local governments’ fiscal capacities and give local governments with low fiscal self-sufficiency more fiscal resources.

The remainder of this paper is organized as follows. Section “Methods” provides the theoretical explanation and hypotheses. Section “Results” describes the construction of the threshold model and data origin. Section “Discussion” presents the empirical results. A summary of findings and their implications for policy and future research are presented in Section “Conclusions”. Conclusion are given in the last section.

### Theoretical explanation and hypotheses

#### The reason for the inter-provincial inequality of public health services and the inefficiency of fiscal transfer payment in China

At the end of 1978, China made the decision to carry out the reform and opening-up policy, and then shifted the focus of government’s work from class struggle to economic development. Instructed by this central policy, economic development is being taken as the governments’ central task and becoming the most important part of the performance evaluation for local officials. According to *Political Promotion Tournament* theory, local party and government leaders are supposed to spare no effort in promoting regional economic development in their short five-year term in order to achieve a higher position under current cadres’ evaluation system [33, 34]. On the other hand, China’s local governments or the local party and government leaders hold the fiscal power, which means that the local party and government leaders have significant leeway to determine the flow of fiscal resources within the existing budget framework. As a result, the majority of fiscal resources are highly like to be used on commercial development projects which can effectively boost GDP in the short-term, or even some *image projects* and *achievement projects* which can draw attentions and show off local governments’ economic achievements. In this case, there is little attention on projects related to people’s livelihoods including public health services. But how could local governments find sufficient and continuous economic development funds to support their political promotion tournament?

Then the phenomenon of embezzlement of fiscal transfer payments occurred. Fiscal transfer payment system was designed to be a useful approach to balance the inter-provincial disparities in public services including public health services. Under this purpose, China’s central government spends huge sums of transfer payments every year, but research performed by most scholars suggests that China’s current fiscal transfer payment system is inefficiency in achieving inter-provincial public services equalization [45–47]. In political promotion tournament, local government leaders spare no effort in promoting regional economic development to win the higher positions. However, fostering regional economic growth is difficult for local leaders without large public capital investments [48]. As a result, the misappropriation happens. In fact, because of the substantial information asymmetries and the lack of effective oversight in the *principal-agent* relationships between central government and local governments in China, fiscal transfer payments can easily be misappropriated by local governments [49].

This embezzlement of fiscal transfer payments under political promotion tournament becomes a common phenomenon, but it is more likely to occur in undeveloped provinces for the reason that economic situation is not satisfactory and the fiscal self-sufficiency are relatively lower there. Undeveloped provinces, on the one hand, suffer an unsatisfactory economic situation and need public capitals to develop economic, but on the other hand are trapped by a relatively low level of self-sufficiency. And this dilemma finally causes the issues on equalization of public health services. Undeveloped provinces have no ability to invest significant amounts of public capital in regional economic development with their own fiscal resources, so the amount of transfer payment funds that are actually used for public health services has been misappropriated for regional economic development while public health services become worse. On the contrary, in developed provinces, the high level of economic development makes the *promotion-achievement effects* (results come from *Political Promotion Tournament*) less prominent, so the embezzlement of fiscal transfer payments is less likely to occur. And cadres’ in developed provinces may even regard people’s livelihoods including the public health services as the next *achievement highlight* for their political promotion. And apart from the level of economic development, even if without the transfer payments funds from central government, the relatively higher fiscal self-sufficiency in developed provinces is able to maintain a quite high level of basic public services by their own fiscal resources. Additionally, although the *principal-agent* relationships between central government and local governments may weaken the supervision function, the governments in developed provinces are more transparent affected by factors such as information technology and education level etc., and misappropriations of transfer payments funds are relatively less rampant. In this situation, the undeveloped provinces faced with a shortage of capital input to public health services while the developed provinces increase those kinds of fund investment. Consequently, the inter-provincial public health services gap between developed provinces and undeveloped provinces is widening year by year even with the huge sums of transfer payments, which means the fiscal transfer payments form used in China is ineffective in promoting the inter-provincial public health services equalization.

**Fig. 1:**
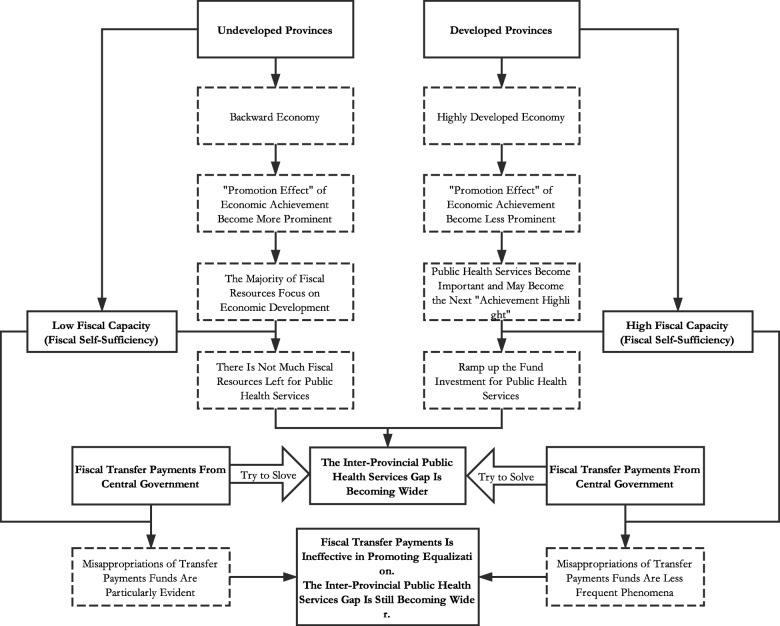
The theoretical analysis flow chart

The previous theoretical analysis flow chart shown clearly in Fig. 1. In conclusion, the local officials’ fanatical pursuit of local economic growth which driven by the “*Political Promotion Tournament*” and the polarized fiscal self-sufficiency of local governments are responsible for the inter-provincial inequality of public health services and the inefficiency of fiscal transfer payment in China.

### The solution to solve the inter-provincial inequality in public health services in countries such as China

What is the solution to the inter-provincial health services inequality in countries such as China? There may be two solutions we can consider: either reform the officials’ performance assessment system to eliminate the negative effect of *Political Promotion Tournament,* or adjust the fiscal self-sufficiency level of local governments to enhance the efficiency of fiscal transfer payments in achieving equalization. China, as a developing nation, still has to give priority to lifting its economic development, which is a principal pillar of the legitimacy of Chinese Communist Party, the ruling party of China [50]. Obviously, the GDP growth rate will still be a critical performance evaluation indicator for local officials for quite a long time. Thus, it seems that we can only consider adjusting the fiscal self-sufficiency level of local governments. From what has been discussed above, we know that it is the polarized fiscal self-sufficiency of local governments that produces the low efficiency of fiscal transfer payments in promoting the inter-provincial public health services equalization. Therefore, adjusting the local governments’ self-sufficiency to the optimal level may be the key to make the fiscal transfer payment system work.

Then, what is the optimal level of the local governments’ fiscal self-sufficiency? We consider that the relationships among local government’s fiscal self-sufficiency, fiscal transfer payments and inter-provincial public health services equalization can be described in three stages.

Stage 1. if a local government’s fiscal self-sufficiency is below the optimal level, it cannot invest significant amounts of public capital in regional economic development by using only its own fiscal resources. In this situation, public health transfer payments are misappropriated and used to promote regional economic growth. Obviously, achieving inter-provincial equalization of public health services is difficult for the fiscal transfer payments under this circumstance.
Stage 2. with the increasing of local fiscal self-sufficiency, on the premise of meeting the need of regional economic growth, a greater amount of fiscal transfer payment funds is available for public health services. In this stage, the efficiency of fiscal transfer payments in promoting inter-provincial public health services equalization gradually increases.
Stage 3. if a local government’s fiscal self-sufficiency exceeds the optimal level, the local governments can already provide high levels of public health services by using their own fiscal resources. Transfer payments from the central government will further improve the high level of public health services. As a result, the gap between these areas and undeveloped areas increases. Therefore, when the fiscal self-sufficiency of local governments reaches a very high level, fiscal transfer payments may become ineffective at promoting inter-provincial public health services equalization.

**Fig. 2:**
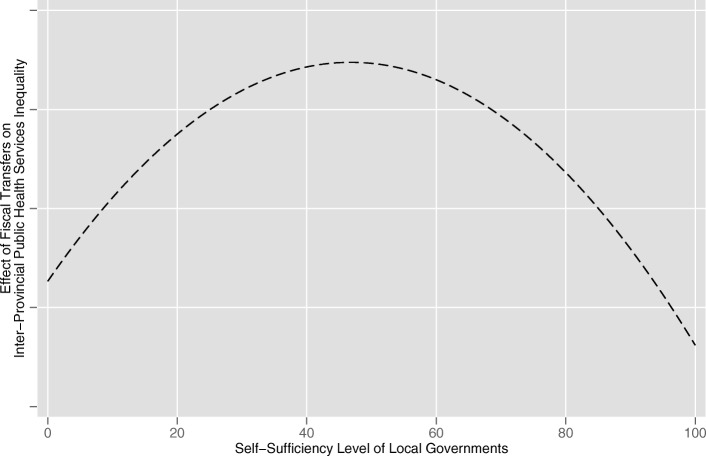
The anticipated effects of fiscal transfer payments on inter-provincial public health services equalization

The anticipated effects of fiscal transfer payments on inter-provincial public health services equalization are as depicted in Fig. 2.

### Hypotheses

Based on the comprehensive analysis presented above, this paper accordingly offers three theoretical hypotheses:

1. *There exist threshold effects between fiscal transfer payments and inter-provincial public health services equalization.*
2. *The effect of fiscal transfer payments on public health services equalization shows a trend that first increases and then decreases as local governments’ fiscal self-sufficiency is enhanced.*
3. *There exist a range or a level of fiscal self-sufficiency for local governments at which the fiscal transfer payments can effectively promote the inter-provincial public health services equalization.*

## Methods

### Panel threshold model

The foregoing analysis suggests that there may be multi-threshold effects between fiscal transfer payments and inter-provincial public health services equalization. And the important goal of the present study is to test whether there exists an optimal level of fiscal self-sufficiency for local governments at which the fiscal transfer payments can effectively promote the inter-provincial public health services equalization. To achieve this goal, this paper applies panel threshold regression model to observe the balanced panel data to find the optimal fiscal self-sufficiency level. The panel threshold model developed by Hansen [51, 52] is effective in capturing the non-linear structural changes and has been widely used in many areas of social science thus fits our purpose well.

Based on the foregoing analysis, we use the fiscal self-sufficiency of local governments (*self*) as the threshold variable and investigate the relationship between the fiscal transfer payments (*transfer*) and the inter-provincial equalization of public health services (*equalization*). In addition, after careful consideration, we introduce indices of economic growth (*gdpg*), educational attainment (*education*), age composition (*dependency*), urbanization level (*urbanization*) and population growth (*population*), which may influence the public health services equalization, as the control variables to strengthen the reliability of our empirical results. Finally, the panel threshold regression model is constructed as follows:

1 $$ {\displaystyle \begin{array}{l}{equalization}_{it}={\alpha}_1^{\hbox{'}}{transfer}_{it}I\left({self}_{it}<{\gamma}_1\right)+{\alpha}_2^{\hbox{'}}{transfer}_{it}I\left({\gamma}_1\le {self}_{it}<{\gamma}_2\right)+\dots \\ {}+{\alpha}_{n+1}^{\hbox{'}}{transfer}_{it}I\left({self}_{it}\ge {\gamma}_n\right)+{\beta}^{\hbox{'}}{X}_{it}+{\mu}_i+{\varepsilon}_{it}\end{array}} $$

Where *I*(⋅) is the indicator function, *X*_*it*_ is a vector of control variables that contains the five variables: *gdpg*, *education*, *dependency*, *urbanization* and *population*. An alternative intuitive way of writing (1) is:

2 $$ {equalization}_{it}=\left\{\begin{array}{c}{\alpha}_1^{\hbox{'}}{transfer}_{it}+{\beta}^{\hbox{'}}{X}_{it}+{\mu}_i+{\varepsilon}_{it}\\ {}{\alpha}_2^{\hbox{'}}{transfer}_{it}+{\beta}^{\hbox{'}}{X}_{it}+{\mu}_i+{\varepsilon}_{it}\\ {}\dots \\ {}{\alpha}_{n+1}^{\hbox{'}}{transfer}_{it}+{\beta}^{\hbox{'}}{X}_{it}+{\mu}_i+{\varepsilon}_{it}\end{array}\right.{\displaystyle \begin{array}{c} if\;{self}_{it}<{\gamma}_1\\ {} if\;{\gamma}_1\le {self}_{it}<{\gamma}_2\\ {}\dots \\ {} if\;{self}_{it}\ge {\gamma}_n\end{array}} $$

### Measurement

#### Dependent variables

The dependent variable is the utility of inter-provincial equalization of public health services (*equalization*). We can calculate the *equalization* of province *i* in the year *t* with the following the relative deviation formula (3):

3 $$ {equalization}_{it}=-\left|\frac{PHS\; level\kern0.17em of\kern0.17em province\;i\; in\kern0.17em year\;t- national\kern0.17em average\kern0.17em level\kern0.17em in\kern0.17em year\;t}{national\kern0.17em average\kern0.17em level\kern0.17em in\kern0.17em year\;t}\right| $$

From formula (3), we can see that *equalization* can only be negative or zero, and it represents the gap between the provincial level and the national average for public health services. The higher the number, the higher the equality degree. To test the reliability of the empirical results, this paper uses two methods to measure the public health services (PHS) level by following the previous research [3, 5, 39, 53–55]*.* They are, the number of beds in health care institutions per 1000 population and the number of nurses in health care institutions per 1000 population, corresponding to Model 1 and Model 2, respectively. In fact, due to the changing of the statistical scope, the number of beds in health care institutions per 1000 population and the number of nurses in health care institutions per 1000 population are the most comparable data we can get. Thus, this paper conducts a comprehensive comparative analysis by using two different methods and attempts to reduce errors by cross-referencing.

#### Threshold variable and core independent variable

The threshold variable is the fiscal self-sufficiency of local government (*self*). The core independent variable is the level of transfer payments (*transfer*). Adopting a common approach, this paper uses the ratio of local government general budgetary revenues to local general budgetary expenditures to measure the value of *self.* To measure *transfer*, we use the absolute amounts of fiscal transfer payment funds for each local government.

#### Control variables

The exogenous control variables are economic growth (*gdpg*), educational attainment (*education*), age composition (*dependency*), urbanization level (*urbanization*) and population growth (*population*), which are measured as the actual growth rate of per capita GDP, the number of college graduates per 1000 population, the dependency ratio of population, the percentage of urban population occupying the total population and the provincial natural population growth rate, respectively.

### Data collection

The sample data cover 31 provincial-level administrative regions (Hong Kong, Macao, and Taiwan are excluded). The period of the data starts in 1997, when the transfer payment system was basically completed, and spans 19 years, ending in 2015. All basic data are derived from the *China Statistical Yearbooks from 1998 to 2016*, *Finance Yearbook of China from 1998 to 2016*, *China Health and Family Planning Yearbook from 2003 to 2016* and *China Health Statistics Yearbook from 1998 to 2013*. Note that GDP covered by this paper are corrected against the 1996 baseline by using the GDP deflator. The descriptive statistics for the variables used in this study are summarized in Table 1.

**Table 1:** Descriptive statistics

Variable	Observations	Mean	SD	Min	Max
*equalization1 (%)*	589	−24.128	31.539	− 171.983	0
*equalization2 (%)*	589	−35.115	49.226	− 260	0
*self (%)*	589	51.705	19.741	5.303	95.086
*transfer (10 billion yuan)*	589	7.108	7.232	0.158	37.796
*gdpg (%)*	589	11.196	2.554	3	23.8
*education (per 1000 population)*	589	76.939	59.480	0.905	423.35
*dependency (%)*	589	39.330	7.969	19.27	64.49
*urbanization (%)*	589	46.511	16.192	16.9	89.6
*population (‰)*	589	5.941	3.327	−1.8	16

## Results

### Panel unit root tests and panel cointegration tests

To avoid spurious regression and guarantee our test can truly reflect the equilibrium relationship between dependent variable and explanatory variable, we have to test whether the time series in this study are stationary or not. We use four panel unit root tests in this study, which include Levin-Lin-Chu testing method proposed by Levin et al. [56], Im, Pesaran and Shin testing method proposed by Im et al. [57], ADF-Fisher testing method proposed by Maddala and Wu [58] and Pesaran’s simple panel unit root testing method proposed by Pesaran [59]. Table 2 shows that no single variable can pass all four tests, suggesting that the variables at level are found to have panel unit roots. So we do first-order differentiation to eliminate unit roots, and under which all variables are given stationary characteristics.

**Table 2:** Panel unit root tests

		Levin-Lin-Chu test	Im, Pesaran and Shin test	ADF-Fisher test	Pesaran’s simple test
Levels	*equalization1*	1.668	4.625	40.423	0.872
	*equalization2*	3.438	5.394	23.514	2.475
	*self*	−5.815^**^	−3.216^**^	176.639^**^	1.238
	*transfer*	10.398	13.971	0.976	−0.946
	*gdpg*	−1.498	−1.197	54.575	−1.602
	*education*	−4.436^**^	−2.585^**^	4.094	−6.582^**^
	*dependency*	−2.617^**^	−1.444	78.900	−2.087^*^
	*urbanization*	−0.949	3.306	79.842	−1.582
	*population*	−4.032^**^	−0.756	167.540^**^	−0.905
Difference	*d_equalization1*	−6.984^**^	−6.54^**^	192.482^**^	−2.908^**^
	*d_equalization2*	−5.027^**^	−6.804^**^	249.782^**^	−6.042^**^
	*d_self*	−7.563^**^	−7.729^**^	169.105^**^	−3.514^**^
	*d_transfer*	−4.195^**^	− 3.157^**^	60.952^**^	− 3.805^**^
	*d_gdpg*	−9.191^**^	−10.531^**^	238.575^**^	−4.769^**^
	*d_education*	−12.749^**^	−14.756^**^	354.954^**^	−10.993^**^
	*d_dependency*	−11.878^**^	−12.309^**^	190.559^**^	−7.640^**^
	*d_urbanization*	−5.895^**^	−5.769^**^	166.369^**^	−4.812^**^
	*d_population*	−6.956^**^	−8.986^**^	170.687^**^	−7.907^**^

Note: “**” and “*” indicate significance levels of 1 and 5%, respectively

Since all the variables are found to be stationary at their first difference level, we use cointegration test to check long-term equilibrium relationship between variables. Specifically, we apply Pedroni testing method and Westerlund testing method. For comparison purposes, this paper uses two methods to measure the utilities of inter-provincial public health services equalization. They are corresponding to Model 1 and Model 2, respectively. As shown in Table 3, both models have cointegration relationship. Therefore, we conclude that the *equalization1* and *equalization2* both exhibit long-term equilibrium relationship between the variables under study, which also means the following threshold test on our panel data can make sense.

**Table 3:** Panel cointegration tests

	Model 1 Dependent variable: *equalization1*		Model 2 Dependent variable: *equalization2*	
	Statistics	*p*-value	Statistics	*p*-value
*Pedroni test*				
Modified Phillips-Perron t	7.122^**^	0.000	5.861^**^	0.000
Phillips-Perron t	−9.080^**^	0.000	−16.000^**^	0.000
Augmented Dickey-Fuller t	− 7.601^**^	0.000	−13.295^**^	0.000
*Westerlund test*				
Variance ratio	−1.647^*^	0.050	−1.932^*^	0.027

Note: “**” and “*” indicate significance levels of 1 and 5%, respectively

### Tests of threshold effect

We firstly tested the existence of threshold effect between fiscal transfer payments and inter-provincial public health services equalization for 31 provinces. We estimated the number of thresholds, allowing for zero, one, two and three thresholds. The bootstrap method was used to obtain an approximation of the F-statistics and then calculate the *p*-values [60]. For each of the three bootstrap tests, 1000 bootstrap replications were used. Table 4 presents the empirical results of the test for single threshold, double threshold and triple threshold effects. As indicated in Table 4, the test statistic strongly rejects the linear model. We find that the test for the single threshold, double threshold and triple threshold are all highly significant. The tests for triple threshold are significant at 1% level in both Model 1 and Model 2, with bootstrap p-value of 0.004 and 0.000, respectively. Thus, we conclude that there is very strong evidence that there are three thresholds in the relationship between fiscal transfer payments and inter-provincial public health services equalization.

**Table 4:** Tests for threshold effects between fiscal transfer payments and inter-provincial public health services equalization

Model	Test	*F*- statistics	*p*-value	Critical values
Model 1	Single threshold	9.881^**^	0.000	(2.587, 3.892, 6.381)
	Double threshold	12.826^**^	0.001	(2.688, 3.924, 6.495)
	Triple threshold	7.686^**^	0.004	(2.864, 3.979, 6.610)
Model 2	Single threshold	24.616^**^	0.000	(2.659, 3.695, 6.915)
	Double threshold	23.660^**^	0.000	(−0.679, 1.817, 6.755)
	Triple threshold	9.482^**^	0.000	(2.787, 3.981, 7.023)

Note: (1) *F*-statistics and *p*-values are derived by using the bootstrap method with 1000 repeats. (2) “**” indicates significance level of 1%

The point estimates of the thresholds and the corresponding 95% confidence intervals are reported in Table 5. The estimates of the three thresholds are 29.236, 43.765 and 63.248 in Model 1, and 28.575, 45.746 and 79.759 in Model 2. The three thresholds separate the range of fiscal self-sufficiency of local government (*self*) into four regimes. It is worth noting that the confidence intervals for thresholds are reasonably tight and the point estimates of the first and second thresholds in Model 1 and Model 2 are quite close to each other, indicating that Model 1 and Model 2 are stable and provide mutual confirmation for each other. The above results strongly confirm our Hypothesis 1.

**Table 5:** Threshold estimates and confidence intervals

Model	Test	Threshold estimates	95% confidence interval
Model 1	Single threshold	43.765	(41.123, 44.756)
	Double threshold	29.236	(25.273, 35.510)
		43.765	(42.444, 44.426)
	Triple threshold	29.236	(25.273, 35.180)
		43.765	(41.123, 44.426)
		63.248	(59.285, 72.824)
Model 2	Single threshold	29.236	(25.273, 31.217)
	Double threshold	28.575	(25.273, 30.887)
		79.759	(75.796, 80.089)
	Triple threshold	28.575	(26.594, 30.226)
		45.746	(44.426, 47.067)
		79.759	(75.796, 80.089)

### Panel threshold regression estimates

Table 6 report the estimation results for the panel threshold model, corresponding to Model 1 and Model 2, respectively. By comparing the results of two models, we get some interesting findings.

**Table 6:** Empirical results of panel threshold regression

		Coefficient	Std. Err.	T	*p* > |t|
Model 1	Dependent variable: *equalization1*				
	Core independent variable				
	*transfer*×*I*(*self* < 29.236)	−0.590^*^	0.237	−2.486	0.013
	*transfer*×*I*(29.236 ≤ *self* < 43.765)	0.253^*^	0.124	2.049	0.041
	*transfer*×*I*(43.765 ≤ *self* < 63.248)	−0.111	0.137	−0.808	0.419
	*transfer*×*I*(*self ≥* 63.248)	−0.649^**^	0.240	−2.707	0.007
	Control variables				
	*gdpg*	−0.323	0.182	−1.779	0.076
	*education*	0.357^**^	0.019	18.434	0.000
	*dependency*	0.259^*^	0.123	2.113	0.035
	*urbanization*	−0.855^**^	0.150	−5.719	0.000
	*population*	−0.814^**^	0.316	−2.573	0.010
Model 2	Dependent variable: *equalization2*				
	Core independent variable				
	*transfer*×*I*(*self* < 28.575)	−1.418^**^	0.312	−4.545	0.000
	*transfer*×*I*(28.575 ≤ *self* < 45.746)	0.310^*^	0.133	2.328	0.020
	*transfer*×*I*(45.746 ≤ *self* < 79.759)	−0.073	0.185	−0.396	0.693
	*transfer*×*I*(*self ≥* 79.759)	−1.505^**^	0.369	−4.085	0.000
	Control variables				
	*gdpg*	−0.018	0.205	−0.087	0.931
	*education*	0.334^**^	0.021	15.773	0.000
	*dependency*	0.220	0.137	1.603	0.110
	*urbanization*	−0.771^**^	0.168	−4.594	0.000
	*population*	−1.316^**^	0.343	−3.830	0.000

Note: “**” and “*” indicate significance levels of 1 and 5%, respectively

Five control variables in Model 1 and Model 2 show the similar effects on inter-provincial public health services equalization. *Education*, *urbanization* and *population* are always significant at 1% level in both Model 1 and Model 2. Educational attainment (*education*) positively affect the inter-provincial public health services equalization, indicating the importance of improving the quality of education. Both urbanization level (*urbanization*) and population growth (*population*) have a negative role to play in inter-provincial public health services equalization, due to the fact that a large number of rural populations in undeveloped provinces have flocked to the big cities in developed provinces in recent decades.

**Fig. 3:**
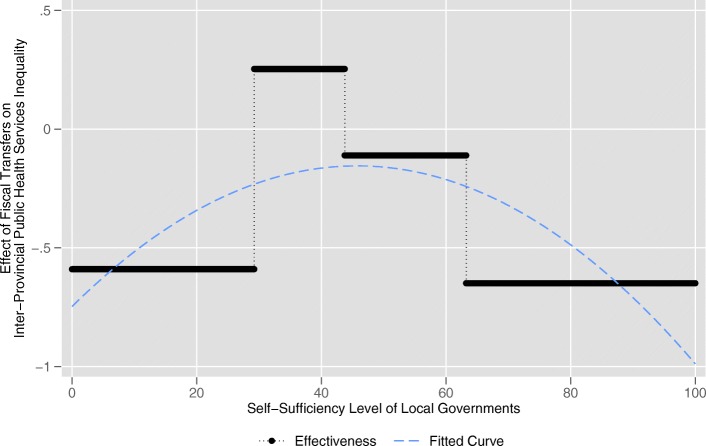
The actual effects of fiscal transfer payments on inter-provincial public health services equalization (Model 1)

**Fig. 4:**
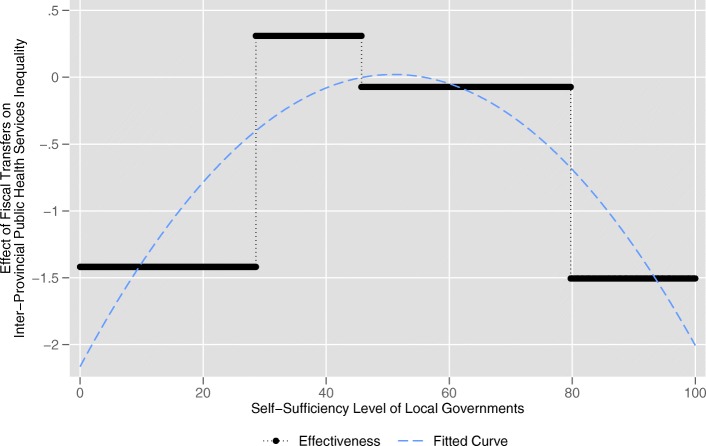
The actual effects of fiscal transfer payments on inter-provincial public health services equalization (Model 2)

Turning to the variable of interest to us, the fiscal transfer payments, the results of Model 1 show that fiscal transfer payments (*transfer*) is significantly and negatively (− 0.590) related to the inter-provincial public health services equalization (*equalization1*) at the 5% significance level when fiscal self-sufficiency of local government (*self*) is less than 29.236%. For provinces with *self* greater than 29.236% and less than 43.765%, the relationship between *transfer* and *equalization1* is significantly (5% level) positive (0.253). The effect becomes insignificantly negative (− 0.111) when *self* lies between 43.765 and 63.248%. After that, with *self* exceeds 63.248%, the relationship between *transfer* and *equalization1* becomes significantly (1% level) negative (− 0.649). The similar results can be also observed in Model 2, when *self* is lower than 28.575%, *transfer* has a significantly (1% level) negative (− 1.418) impact on *equalization2*. When *self* is greater than 28.575% and less than 45.746%, the relationship between *transfer* and *equalization2* becomes significantly (5% level) positive (0.310). When *self* lies between 45.746 and 79.759%, the relationship is insignificantly negative (− 0.073). When *self* exceeds 79.759%, the effect of *transfer* on *equalization2* becomes significantly (1% level) negative (− 1.505). According to the results of Model 1 and Model 2, we create Fig. 3 and Fig. 4, respectively. We can clearly see that the effects of fiscal transfer payments on the inter-provincial public health services equalization in both figures present the trend that first increase and then decrease as the fiscal self-sufficiency of local governments increases which is consistent with our Hypothesis 2. The effect in Model 1 reach its maximums positive when *self* lies between 29.236 and 43.765% and Model 2 is between 28.575 and 45.746%. The intervals are quite close. That is to say, the fiscal transfer payments can effectively promote inter-provincial equalization under the circumstance that fiscal self-sufficiency level of local government is between 29.236 and 43.765% or between 28.575 and 45.746%. The result is consistent with our Hypothesis 3.

In conclusion, both Model 1 and Model 2 show that there exist threshold effects between fiscal transfer payments and inter-provincial public health services equalization, the impact of fiscal transfer payments on inter-provincial public health services equalization first increases and then decreases with the advancing of the local governments’ fiscal self-sufficiency level, and there exist a range of fiscal self-sufficiency for local governments at which the fiscal transfer payments can effectively achieve inter-provincial public health services equalization. The results of Model 1 and Model 2 are similar and can be used to support each other. The empirical results are so stable and reliable that our three hypotheses are well-verified.

### Further analysis

**Fig. 5:**
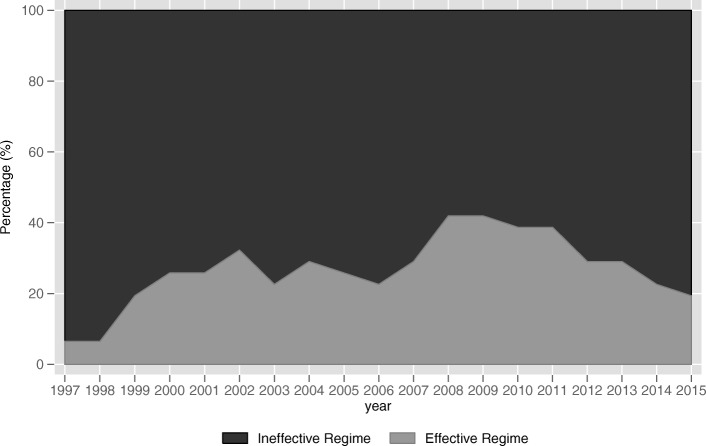
Percentage of provinces in each regime by year (Model 1)

**Fig. 6:**
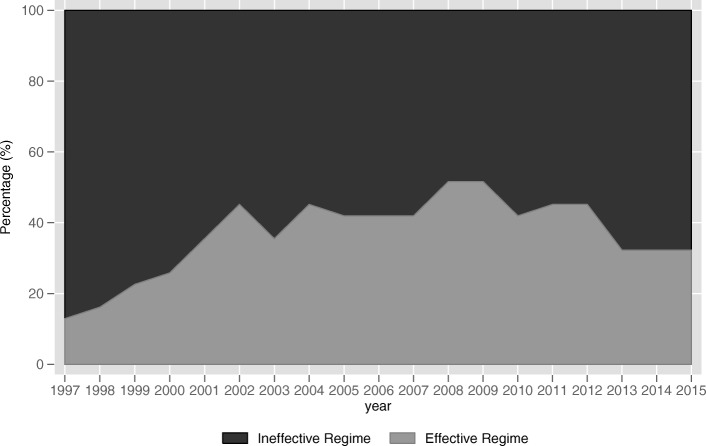
Percentage of provinces in each regime by year (Model 2)

According to the thresholds and different effectiveness of fiscal transfer payments on achieving inter-provincial public health services equalization in Model 1, China’s 31 provincial administrative regions can be divided into two regimes, the effective regime (29.236 ≤ *self* < 43.765) and the ineffective regime (*self* < 29.236, 43.765 ≤ *self* < 63.248, and *self* ≥ 63.248). Figure 5 reports the percentage of provinces which fall into the two regime each year from 1997 to 2015. Similarly, China’s 31 provincial administrative regions can also be divided into two regimes based on the results of Model 2, the effective regime (28.575 ≤ *self* < 45.746) and the ineffective regime (*self* < 28.575, 45.746 ≤ *self* < 79.759, and *self* ≥ 79.759). Figure 6 reports the percentage of provinces in each regime for each year from 1997 to 2015.

From Fig. 5 and Fig. 6, we can clearly see that the vast majority of provinces remain in the ineffective regime at which the fiscal transfer payment is inefficiency in promoting inter-provincial public health services equalization, leading to the inefficiency of China’s current inter-governmental transfer payment system. Overall, the number of provinces in the effective regime show an increase in the 19 years, but still remains a tiny part of the total provinces. It is interesting to note that the number of provinces in the effective regime suffers significant reductions after the year of 2012. We think the reason lies in that Xi Jinping strengthened the fiscal centralization in order to consolidate his power base after coming to power. In conclusion, it is crucial to adjust the fiscal self-sufficiency of local governments to promote the efficiency of fiscal transfer payments in promoting inter-provincial equalization of public health services.

## Discussion

This paper argues that the local officials’ fanatical pursuit of local economic growth driven by *Political Promotion Tournament* and the polarized fiscal self-sufficiency level of local governments are responsible for the inefficiency of fiscal transfer payments and the inter-provincial inequality of public health services in China. Based on theoretical analysis, this paper suggests that we can adjust local governments’ self-sufficiency level to enhance the effectiveness of fiscal transfer payments in achieving equalization of provincial public health services Then, this paper constructs panel threshold regression models with fiscal self-sufficiency of local governments as the threshold variable, and try to investigate the optimal level of local governments’ self-sufficiency where fiscal transfer payments can work effectively for equalization of provincial public health services. The statistic results suggest that threshold effects exist between fiscal transfer payments and inter-provincial public health services equalization, and those effects on equalization of public health services first increase and then decrease as the fiscal self-sufficiency of local governments increases. Specifically, there exist a range of fiscal self-sufficiency either between 29.236 and 43.765% or between 28.575 and 45.746% for local governments that fiscal transfer payments can work effectively to achieve inter-provincial public health services equalization. Our further analysis shows that in most provinces in China, fiscal transfer payments still play an inefficient role, and it is inefficient at least in shaping the disparities on provincial public health standards. Therefore, adjusting the financial capacity of local governments has become an urgent task.

Adjusting the local government’s fiscal capacities means reforming the current tax-sharing financial system. To fully understand China’s current financial system, we should look back into the history of tax-sharing reform started in 1994. Before the tax-sharing reform, the Chinese local governments held the powers of collecting principal tax and the central government shared a certain proportion of tax revenue. Under the old financial system, the local governments lose the motivation to increase tax revenue or even hid the tax revenue because they were reluctant to share their revenues with central government. The consequences were serious. The fiscal revenues of central government did not keep pace with the rapid growth of Chinese economy, the fiscal revenue accounted for only 14% of GDP in 1992, while the proportion in 1978 when the economic reform began was 31% [61]. In 1992, only 28% of fiscal revenue was in the hands of central government [62], which led to a serious fiscal crisis of central government. The disequilibrium of central government’s political power and financial power has greatly weakened central government’s ability to regulate micro-economy and controlling power over local governments. To solve the problem, China began to implement the reform of its tax-sharing system in 1994. The tax was divided into national tax, local tax and shared tax. 75% of shared tax and national tax, the great part of the main taxes, were collected by the National Taxation Administration controlled by central government, while 25% of shared tax and local tax were collected by Local Taxation Bureau of local governments. By taking this measure, the central government successfully controlled the primary source of revenue and had enough fiscal resources to build up mammoth infrastructure programs such as high-speed rail, national expressway network and large-scale water projects.

The tax-sharing reform indeed greatly promoted China’s economic development, and the fiscal capacity of Chinese central government has already been reinforced fully over the past 25 years. However, the broad-brush tax-sharing caused the polarization of local governments’ fiscal capacities. The local governments in undeveloped provinces are faced with serious financial difficulties, while the local governments with relatively high fiscal self-sufficiency in developed provinces have enjoyed high economic growth due to the huge tax rebates from central government. Hence, the provincial fiscal gap widened. Moreover, the local governments in undeveloped provinces borrowed heavily because of the shortage of capital. By the end of 2012, the local governments’ debts, at about 9.6 trillion, was nearly 1.67 times of local governments’ revenues (not including fiscal transfer payments) and still rapidly rising [63]. The extreme polarization of local governments’ fiscal self-sufficiency has become a serious problem, severely restricting China’s sustainable development and exacerbating the inter-provincial inequality of public health services. Therefore, maybe it is time to reform the tax-sharing financial system and give local governments with low fiscal self-sufficiency more fiscal resources to exercise their powers and fulfill their responsibilities such as providing equitable and almost differentiated public health services.

The optimal range of fiscal self-sufficiency for local governments proposed by this paper has important policy implications for China’s financial system reform and is helpful to policy makers. The policy suggestion from our study is thus very clear. The effective way to narrow the inequality of inter-provincial public health services is to establish a flexible tax-sharing system to adjust local governments’ fiscal capacities. By dividing China’s 31 provincial administrative regions into different regime, the policy makers can clearly see which province we should give more fiscal powers and supports and which province we should weaken its fiscal power.

Actually, the new policy measures recently launched by Chinese central government coincide with our recommendations. On February 08, 2018, China issued a financial reform plan on the distribution of responsibilities and powers among the central and local governments with regard to basic public health services. According to the economic development level and the actual fiscal capacity in each province, China’s central government divided 31 provinces into four regimes. Among them, for the western undeveloped provinces with low fiscal capacities, the central government bears 80% of the basic public health services expenditures. However, for developed provinces including Beijing and Shanghai, the central government only bears 10% of the expenditures. The implementation of this plan will ensure that provinces with low fiscal capacities provide quality basic public health services to local residents.

## Conclusions

This paper explains the reason of inequality in public health services and the inefficiency of fiscal transfer payment system from Chinese local officials’ behavior aspect, and try to find out an effective solution by focusing on the local government’s fiscal capacity. The effective way to narrow the inter-provincial public health services inequality is to establish a flexible tax-sharing financial system to adjust local governments’ fiscal capacities and give local governments with low fiscal self-sufficiency more fiscal resources. The new policy measures recently launched by Chinese central government coincide with our recommendations.

In a broader sense, as has discussed at the beginning, developing countries whose social economic situations are similar with China and intend to solve the problem of inequality in public services, can learn from China case when American model and German model are not suitable on the basis of their current situations. We believe that there will be some difficulties other developing countries will encounter when they choose financial transfer payments as a feasible way to solve the equalization problem of regional public services that has not been discussed in our study, we suggest that further research could start from this view.

## Abbreviations

GDP: Gross domestic product
PHS: Public health services
