# Correction to: Inter-provincial inequality of public health services in China: the perspective of local officials’ behavior

**DOI:** 10.1186/s12939-018-0839-4

**Published:** 2018-09-04

**Authors:** Tianxiang Chen, Ying Wang, Xiaoyi Luo, Yuxuan Rao, Lei Hua

**Affiliations:** 1 School of Government, Sun Yat-sen University, Guangzhou, Guangdong China; 2 Department of Public Administration, Nanfang College of Sun Yat-sen University, Guangzhou, Guangdong China; 3 School of Law and Business, College of Science and Technology of Ningbo University, Ningbo, Zhejiang, China; 4 College of Liberal Art and Science, University of Illinois at Urbana-Champaign, Urbana, Illinois USA

## Correction

Unfortunately, after publication of this article [1], it was noticed that a value in Table 2 is incorrect. In the column ‘ADF-Fisher test’ in the row ‘d_transfer’, the value displayed as 60.952 should instead read 125.781. Subsequent tests and results were based on the latter figure so this change does not affect the results and conclusions drawn.
